# Endophytic bacterium *Pseudomonas protegens* suppresses mycelial growth of *Botryosphaeria dothidea* and decreases its pathogenicity to postharvest fruits

**DOI:** 10.3389/fmicb.2022.1069517

**Published:** 2022-12-08

**Authors:** Yonghong Huang, Junping Liu, Jinghui Li, Xiaoying Shan, Yanxin Duan

**Affiliations:** ^1^College of Horticulture, Qingdao Agricultural University, Qingdao, China; ^2^Laboratory of Quality and Safety Risk Assessment for Fruit, Ministry of Agriculture and Rural Affairs, Qingdao, China; ^3^National Technology Centre for Whole Process Quality Control of FSEN Horticultural Products, Qingdao, China; ^4^Qingdao Key Laboratory of Modern Agriculture Quality and Safety Engineering, Qingdao, China

**Keywords:** biological control, fruit quality, defense-related genes, pathogenicity-related genes, apple ring rot

## Abstract

Apple (*Malus domestica* Borkh.), one of the most economically important fruits widely consumed worldwide, has been suffering from apple ring rot caused by *Botryosphaeria dothidea*, which dramatically affects its quality and yield. In the present study, we demonstrated that *Pseudomonas protegens*, isolated from Chinese leek (*Allium tuberosum*), significantly suppressed the mycelial growth and propagation of *B. dothidea*, respectively, further displayed a considerably inhibitory effect on the apple ring rot of postharvest fruits. In addition, *P. protegens* significantly improved the total soluble solid/titrable acidity (TSS/TA) ratio and soluble sugar/titrable acidity (SS/TA) ratio and drastically maintained the fruit firmness. Further analysis manifested that *P. protegens* substantially induced the defense-related genes such as MdGLU, MdPAL, MdPOD, MdCAL, and transcription factors related to the resistance to *B. dothidea*, including MdWRKY15, MdPUB29, MdMyb73, and MdERF11 in apple fruits. Meanwhile, *P. protegens* considerably restrained the expressions of the pathogenicity-related genes in *B. dothidea*, including the BdCYP450, BdADH, BdGHY, BdATS, Bdα/β-HY, and BdSTR. By inference, *P. protegens* inhibited the apple ring rot on postharvest fruits by activating the defense system of apple fruit and repressing the pathogenic factor of *B. dothidea.* The study provided a theoretical basis and a potential alternative to manage the apple ring rot on postharvest fruits.

## Introduction

Apple (*Malus domestica* Borkh.) is one of the most widely produced and economically important fruits in temperate regions (Chen et al., 2021). China is the largest producer in the world, with harvested areas of 1.91 million hectares (41.01% of the world total) and 40.50 million tons (46.85% of the world total) in 2020 (FAOSTAT, 2022).^1^ In recent years, apple fruit has been among the most widely consumed fruits in the world since they are available the whole year, inexpensive, and convenient to consume. Above all, apple contains a range of nutrients with different biological activities, including polyphenols, vitamins, minerals, lipids, proteins/peptides, and carbohydrates (Koutsos and Lovegrove, 2015; Pollini et al., 2021), which are beneficial for our health, as per the saying, “an apple a day keeps the doctor away” (Davis et al., 2015).

Unfortunately, due to the lack of resistant varieties, continuous cropping, and poor field management, apple orchards in China often suffer from pests and diseases. Apple ring rot, caused by *Botryosphaeria dothidea*, is one of the most destructive apple diseases worldwide, including in China, Japan, South Korea, United States, Australia, and South Africa (Wang et al., 2018). *B. dothidea* infects apple trees, resulting in fruit rot, twig dieback, stem and branch canker, and tree death (Dong and Guo, 2020). Besides, *B. dothidea* often infects the apple fruits during the early growth stages, remains latent, and causes fruit rot during ripening or storage (Yu et al., 2022). The decayed fruit incidence caused by the disease usually ranges between 10 and 20% each year and may reach 70% in seasons with conditions conducive to fungal development (Zhao et al., 2016). According to an investigation, the average occurrence of the disease was as high as 77.6% in 88 apple orchards across seven main apple production areas in China (Guo et al., 2009). Therefore, apple ring rot has seriously impeded the sustainable and healthy development of the apple industry in China.

Currently, chemical fungicides are still the primary strategies for controlling apple ring rot disease (Dai et al., 2017; Song et al., 2018; Fan et al., 2019). However, these fungicides were potentially hazardous to human health, other non-target organisms, and the natural environment. In addition, pathogenic microorganisms may evolve fungicide resistance (Yin and Qiu, 2019). Given the above-described concerns, there is an urgent need to research and develop alternative measures for preventing and controlling the disease.

Biological control is a safe way to control pests and pathogens. Antagonistic microorganisms showed tremendous potential for substituting chemical fungicides to manage plant pathogens (El-Hasan et al., 2017; Fan et al., 2020; Zhao et al., 2021). It is reported that *Pseudomonas* spp. have been widely studied for their biocontrol potential. For example, *P. chlororaphis* significantly reduced the postharvest gray mold on Chinese cherry (Wang C. et al., 2021). *P. fluorescens* drastically suppressed the blue mold disease of postharvest citrus fruits (Wang Z. et al., 2021). *P. segetis* markedly reduced soft rot symptoms in potatoes (Rodriguez et al., 2020). *P. aeruginosa* considerably controlled several pathogens in tomatoes, potatoes, taro, and strawberries (Ghadamgahi et al., 2022). *P. putida* strongly reduced the common bean rust disease severity (Abo-Elyousr et al., 2021). *P. synxantha* significantly reduced brown rot incidence and severity on peaches (Aiello et al., 2019). *P. parafulva* effectively controlled the soybean bacterial pustule (Kakembo and Lee, 2019). In addition, *P. protegens* had intense antifungal activity against various plant pathogens, including *Heterobasidion abietinum*, *Heterobasidion annosum*, *Heterobasidion irregulare*, and *Heterobasidion parviporum* (Pellicciaro et al., 2021), *Calonectria pseudonaviculata* (Yang and Hong, 2018), *Rhizoctonia solani* (Jing et al., 2020), *Fomes fomentarius*, *Ganoderma lucidum*, *Phellinus pini*, *Phellinus tuberculosus*, *Sclerotinia sclerotiorum*, *Alternaria tomatophila* (Prigigallo et al., 2021), *Alternaria alternata*, *Aspergillus niger*, *Penicillium expansum*, *Neofusicoccum parvum* (Andreolli et al., 2019), *Botrytis cinerea*, and *Monilinia fructicola* (Zhang et al., 2020). In our preliminary study, we isolated an endophytic bacterium *P. protegens* from Chinese leek (*Allium tuberosum*). The present study demonstrated the antifungal activity against *B. dothidea* and the inhibitory effect on ring rot on postharvest apple fruits. Further, we primitively uncovered the underlying mechanism from the apple fruit aspect and the pathogen *B. dothidea* aspect to provide a theoretical basis and an alternative for controlling the apple ring rot on postharvest fruits.

## Materials and methods

### Experiment materials

Apple fruits (*Malus domestica*, cv. Fuji) were purchased from the local market. Fruits in uniform size and color, free of visible disease and mechanical injuries, were selected for the experiments. The endophytic bacterium *P. protegens* isolation NSJ-2101 and the pathogen fungus *B. dothidea* isolate LW-1801 were cultured on the nutrient agar (NA) and potato dextrose agar (PDA) medium, respectively, and kept in our laboratory.

### The suppressive effect of the *Pseudomonas protegens* on *Botryosphaeria dothidea* growth

We conducted the experiments using the PDA and potato dextrose broth (PDB) medium.

#### Potato dextrose agar medium experiment

The PDA medium (20 ml) was poured into a 9-cm-diameter Petri dish. One mycelium disc (0.5 cm in diameter) of the fungus *B. dothidea* was inoculated in the center of the Petri dish. Then one disc of *P. protegens* was inoculated on each side 2 cm from the fungal disc (Pp). Sterilized water (50 μL) was used as the control (CK). All the Petri dishes were inverted and incubated at 28°C in the dark for 3 days. The diameters of the fungal colony were measured daily to evaluate the inhibition of *P. protegens* on the mycelial growth *B. dothidea*. After that, *P. protegens*-treated mycelia and the control mycelia were carefully picked from the Petri dishes and observed under a microscope (EVOS Auto2, Thermo Fisher Scientific, United States) with × 40 magnification. The experiments were repeated three times, and five replicates were included for each sample in each experiment.

#### Potato dextrose broth medium experiment

Various amount (2.5, 5, and 10 ml) of *P. protegens* culture (OD_600_ = 0.6) was added into a 150 ml *Erlenmeyer flask*, respectively. PDB medium was appended until it reached a total volume of 50 ml, making the concentrations of the *P. protegens* 5, 10, and 20%. 50 ml of PDB medium was used as a control. Then, one mycelium disc (0.5-cm-diameter) of *B. dothidea* was put into the *Erlenmeyer flask*. The *Erlenmeyer flask* was incubated at a constant temperature shaking incubator at 28°C, shaking at 200 rpm for 2 days. After centrifugation, the precipitate was collected and weighed to evaluate the suppression of *P. protegens* on the propagation of *B. dothidea*. The experiments were performed in triplicates.

### The inhibitory effect of the *Pseudomonas protegens* against the apple ring rot on the postharvest fruit

According to the different ways of inoculating fungus *B. dothidea*, we performed two experiments to test the control effect of *P. protegens* on apple ring rot.

#### Experiment 1

The apple fruits were surface-sterilized with 1.5% NaOCl for 5 min, washed thrice with distilled water, and air-dried. Then a tiny wound (3 mm width × 5 mm deep) was created at the equator in each apple fruit using a sterile needle. Next, 20 μl of *P. protegens* (OD_600_ = 0.6) was added into the small cavity. One hour later, a *B. dothidea* mycelial disc (3 mm in diameter) was inoculated into the wound. Again, 20 μl of the nutrient broth (NB) medium was used as a control. Finally, all the apple fruits were packed into a black plastic box and incubated at 28°C under dark conditions for 3 days. The disease spot diameter was measured to evaluate the inhibitory effect of the *P. protegens* on the apple ring rot incidence on fruits. The experiment was repeated ten times.

#### Experiment 2

First, the apple fruits were surface-sterilized and air-dried, as in experiment 1. Then the apple fruits were immersed in *P. protegens* culture (OD_600_ = 0.6) for 15 min and soaked in *B. dothidea* (OD_600_ = 0.6) culture for another 15 min after they were air-dried at room temperature for 1 h (Pp + Bd). In addition, the apple fruits that were soaked in NB medium for 15 min, air-dried at room temperature for 1 h, then dipped in *B. dothidea* culture for 15 min were used as the control (Bd). Next, all the fruits were packed in plastic bags and incubated at 28°C. Finally, the disease symptom, such as mycelial cluster and disease spots, were observed and recorded on 1, 3, 5, 7, and 9 days to evaluate the inhibitory effect of *P. protegens* on apple ring rot. The experiment was performed in triplicate.

### The effect of *Pseudomonas protegens* on the apple fruit quality

To fully confirm whether *P. protegens* affected the internal quality of apple fruit, we designed four treatments (CK, Pp, Bd, and Pp + Bd). (1) CK: The apple fruits were soaked in PDB medium for 15 min. (2) Pp: The apple fruits were soaked in *P. protegens* culture (OD_600_ = 0.6) for 15 min (Pp). (3) Bd and (4) Pp + Bd were described above. All the fruits were packed in plastic bags and set in an incubator at 28°C. Then, fruits were sampled and peeled 9 days later. Fruit firmness was measured at the fruit’s equator with the FHM-5 fruit hardness tester (Takemura Electric Works Ltd., Tokyo, Japan). The pulp at the fruit equator was sampled to determine other internal quality indexes, including total soluble solid (TSS), soluble sugar (SS), titratable acidity (TA), vitamin C (VC), total soluble solid/titrable acidity (TSS/TA) ratio, and soluble sugar/titrable acidity (SS/TA) ratio. TSS content was determined using a PAL-1 type sugar concentration detector (ATAGO, Japan). SS, TA, and VC were determined using the anthrone colorimetric, NaOH titration, and 2, 6-dichloroindophenol colorimetric, respectively (Yang L. et al., 2020). Three replicates were included for each sample at each sampling time point.

### The effect of the *Pseudomonas protegens* on the defense-related gene in the apple fruits

To determine whether the reduction of the fruit disease symptom was caused by the *P. protegens* induction of the fruit defense system, we detected the expressions of the defense-related genes and the transcription factors in apple fruits. The defense-related genes contained phenylalanine ammonia-lyase (MdPAL; XM_008368428.3), glucanase (MdGLU; XM_029095631.1), peroxidase (MdPOD; XM_029099288.1), and catalase (MdCAT; XM_008375181.3). The transcription factors included MdWRKY15 (XM_008395555.3), apple U-box E3 ubiquitin ligase 29 (MdPUB29; XM_008350166.3), MdMYB73 (XM_008379837.3), and apple ethylene response factor 11 (MdERF11; NM_001301117.1), which were reported to enhance the resistance of apple fruits to *B. dothidea*.

According to previously described methods, the four treatments (CK, Pp, Bd, and Pp + Bd) were performed on apple fruits. All the fruits were packed in plastic bags and incubated at 28°C. One day later, the fruits were sampled and peeled around the equator with a sterilized paring knife. All the samples were stored at –80°C for later qRT-PCR.

### The effect of the *Pseudomonas protegens* on the pathogenic-related gene in the fungus *Botryosphaeria dothidea*

We examined several published pathogenicity-related genes to verify whether the reduced disease symptom in the apple fruits was also associated with the repressed expression of vital gene in *B. dothidea* by *P. protegens*. These genes included cytochrome p450 protein (BdCYP450; BOTSDO04127), alcohol dehydrogenase (BdADH; BOTSDO00477), glycoside hydrolase (BdGHY; BOTSDO02303), aminoacyl-tRNA synthetase (BdATS; BOTSDO01704), alpha/beta hydrolase (Bdα/β-HY; BOTSDO04588) and sugar transporter (BdSTR; BOTSDO09350).

A mycelium disc (0.5 cm in diameter) of *B. dothidea* was inoculated on a layer of sterilized cellophane placed on the newly prepared PDA medium. The Petri dish was incubated at 28°C in an inverted position for 2 days in the dark. Then *P. protegens* (2 ml; OD600 = 0.6) was sprayed on the fungal mycelia (Pp). Sterilized water (2 ml) was used as the control (CK). The Petri dishes continued to be incubated in the same condition. Six hours later, the *P. protegens-treated* and the control mycelia were sampled for qRT-PCR.

### qRT-PCR measurement

The abovementioned gene sequences of the apple and the mycelium were retrieved from the apple genome (ASM211411v1) and the *B. dothidea* genome (ASM1150312v2) that were downloaded from the National Center for Biotechnology Information (NCBI) genome website.^2^ Then, the special primers were designed using Primer Primer 5 according to the respective gene sequences (Supplementary Table 1) and synthesized in Sangon Biotech (Sangon Biotech, Shanghai, China).

The total RNA of the prepared apple and the mycelia samples were extracted using RNAprep Pure Plant Plus Kit [Tiangen Biotech., Beijing, China]. And the cDNA was synthesized using HiScript RIII RT SuperMix for qPCR (CgDNA wiper; Vazyme Biotech, Nanjing, China). qRT-PCR was performed using ABI7500 Thermal Cycler (Applied Biosystems, Foster City, CA, United States) to detect the relative expressions of the genes mentioned above. The MdActin and the BdTubulin were used as the apple’s and mycelia’s internal reference genes, respectively. The relative expression was calculated by 2^–ΔΔCT^ method (Livak and Schmittgen, 2001). The experiment was performed in three replicates.

### Statistical analysis

Analysis of variance (ANOVA) was conducted using SAS 8.0 software (SAS Institute Inc., Cary, NC, United States). A least significance difference test (LSD) was applied to determine the significance between different treatments (*p* < 0.05). Standard errors were calculated for all mean values.

## Results

### *Pseudomonas protegens* significantly suppressed the growth of *Botryosphaeria dothidea*

**Figure 1:**
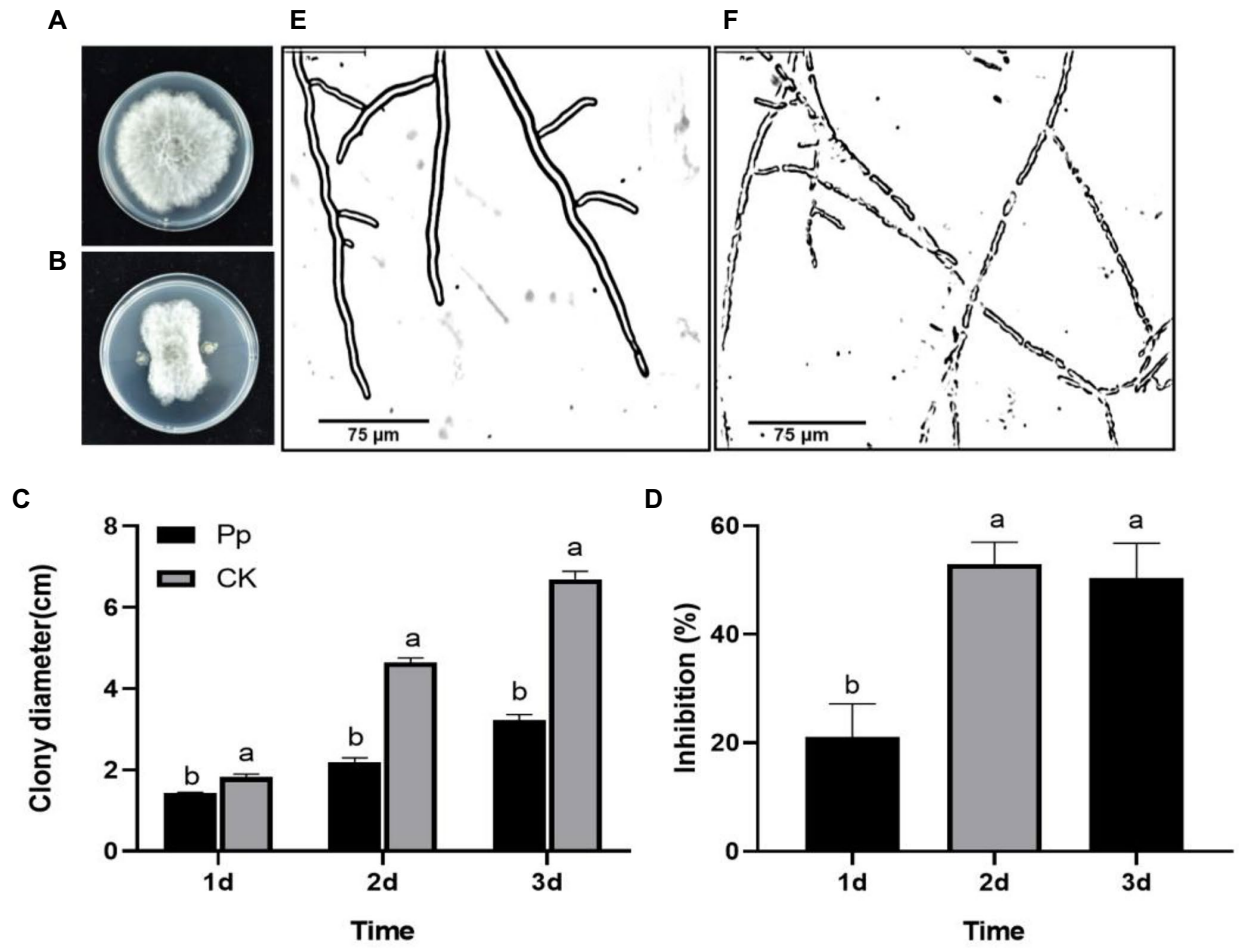
The suppression of *Pseudomonas protegens* on the mycelia growth of *Botryosphaeria dothidea* on the PDA medium. The untreated control mycelia **(A)** and the *P. protegens-*treated mycelia **(B)** that were incubated on the PDA medium for 3 days. The comparison of the colony diameter of the different treatments **(C)**. *P. protegens* significantly inhibited the mycelial growth of the fungus *B. dothidea* **(D)**. Compared to the control **(E)**, the *P. protegens* severely damaged the mycelial morphology of *B. dothidea* **(F)**. Lowercase letters indicate a significant difference between treatments (*p* < 0.05).

On the PDA medium, the new mycelia grew from all the *B. dothidea* discs on the first day. After that, the control mycelia continued to expand rapidly (Figure 1A), but the growth of the *P. protegens*-treated mycelia gradually slowed down (Figure 1B). As a result, the fungal colony diameter treated with *P. protegens* increased to 1.44, 2.19, and 3.23 cm on day 1, day 2 and day 3, respectively (Figure 1C), reduced by 21.08, 52.90, and 50.34% compared to the control (Figure 1D). Microscopic observation demonstrated that the control mycelia was smooth with distinct borders and intact structure (Figure 1E). In contrast, the *P. protegens*-treated mycelia fragmented with blurred boundaries (Figure 1F), which revealed that *P. protegens* damaged the normal mycelial morphology of *B. dothidea*.

**Figure 2:**
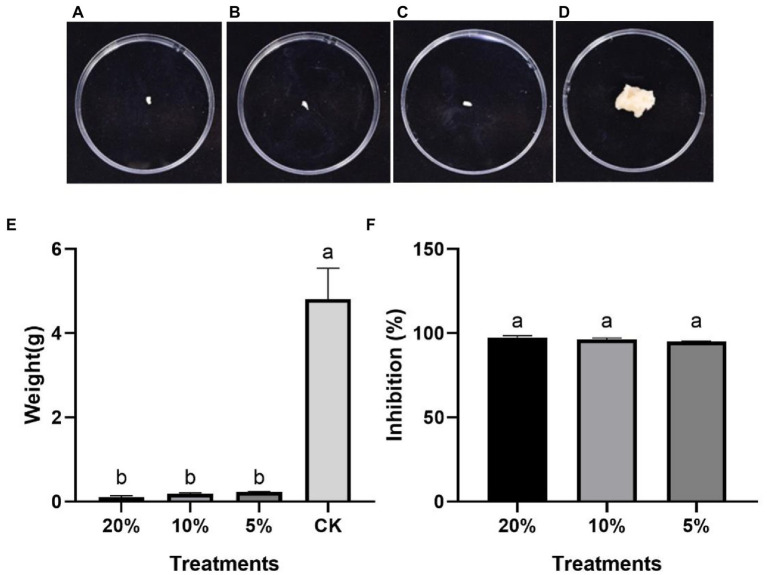
The suppression of *P. protegens* on the mycelia growth of *B. dothidea* in the PDB medium. The mycelia incubated with shaking in the PDB medium supplemented with 20% **(A)**, 10% **(B)**, and 5% **(C)** of *P. protegens* and the control mycelia **(D)** for 2 days. **(E)** The weight of mycelia from different treatments **(E)**. The inhibition of various concentrations of *P. protegens* to the fungus *B. dothidea* **(F)**. Lowercase letters indicate a significant difference between treatments (*p* < 0.05).

On the PDB medium, the *P. protegens*-treated and the control mycelia showed significantly different growth 2 days later. The mycelia treated with various of concentration *P. protegens* showed almost no increase (Figures 2A–C), but the control mycelia developed quickly (Figure 2D). The increased weight of the mycelia treated with 20, 10, and 5% of *P. protegens* was 0.11, 0.19, and 0.24 g (Figure 2E), respectively, which was reduced by 97.62, 96.14, and 95.06%, compared to the control (Figure 2F). It indicated that *P. protegens* significantly suppressed the mycelia growth of *B. dothidea*.

### *Pseudomonas protegens* significantly inhibited the disease severity of apple ring rot on postharvest fruits

#### Experiment 1

**Figure 3:**
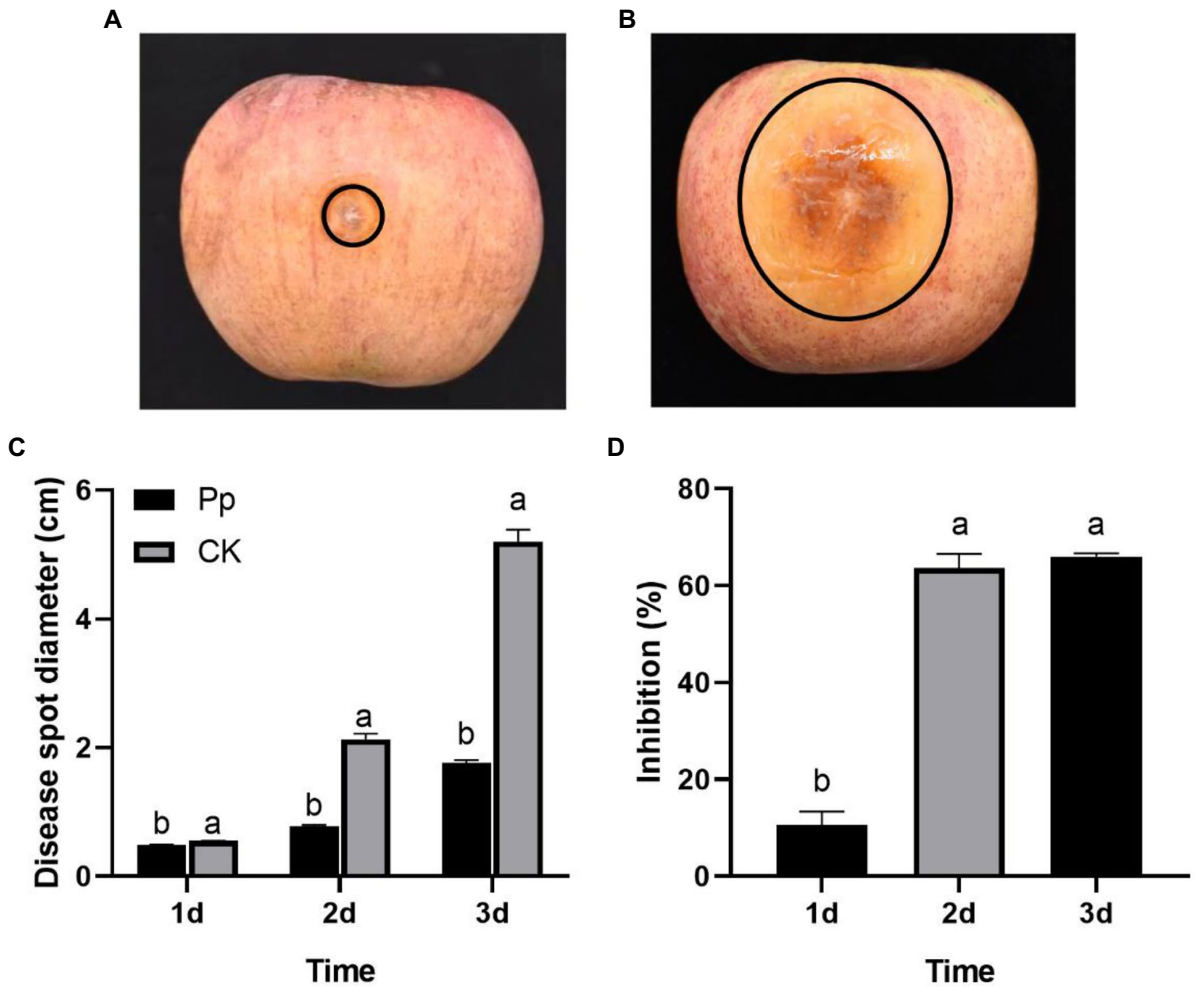
The inhibitory effect of *P. protegens* on apple ring rot on postharvest fruits (Experiment 1). The disease symptom on the *P. protegens-*treated fruits **(A)** and the untreated control fruits **(B)** 3 days later. The disease spot diameter of the *P. protegens-*treated fruits was significantly smaller than the untreated control **(C)**, demonstrating that *P. protegens* had potent inhibition on the ring rot disease on postharvest apple fruits **(D)**. Lowercase letters indicate a significant difference between treatments (*p* < 0.05).

One day later, the disease symptoms appeared at the inoculation sites on the *P. protegens-*treated and the control fruits. And the disease symptoms developed severity over time. However, the disease spots on the *P. protegens*-treated fruits extended slower than the control (Figures 3A,B), the disease spot diameter of which expanded to 0.49, 0.77, and 1.76 cm on day 1, day 2, and day 3, respectively (Figure 3C), decreasing by 10.59, 63.66, and 66.05% compared to the control (Figure 3D).

#### Experiment 2

**Figure 4:**
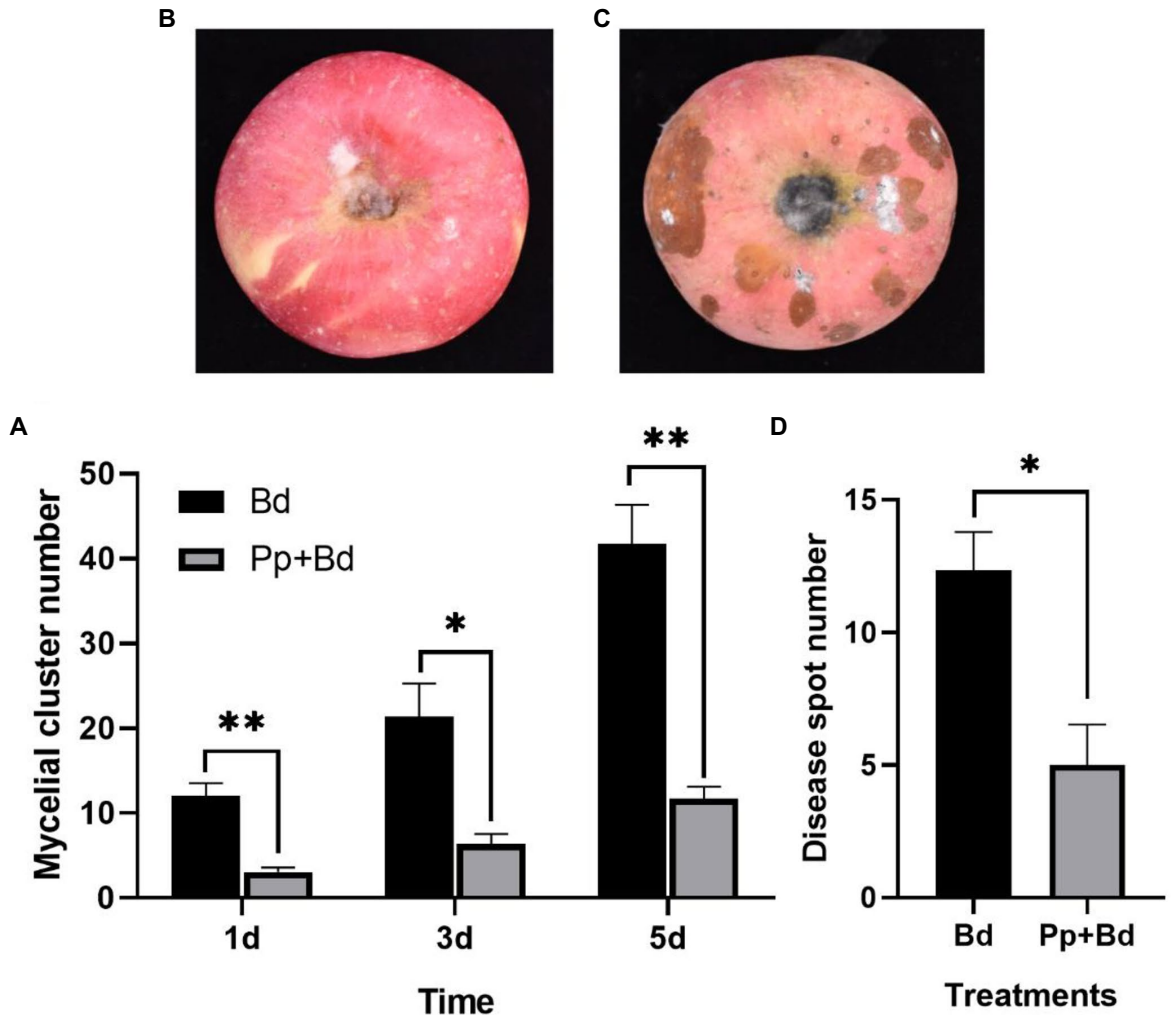
The inhibitory effect of *P. protegens* on apple ring on postharvest fruit (Experiment 2). The mycelial cluster number on *P. protegens*-treated fruits was significantly smaller than the control on the first, third, and fifth day **(A)**. In addition, the disease spots on the *P. protegens-*treated fruits **(B)** were smaller than that on the control fruits on the seventh day **(C)**, and the number of the disease spots on the *P. protegens-*treated fruits was also significantly lower than the control **(D)**. ^*^p  <  0.05, ^**^p   <  0.01.

The greyish-white mycelial cluster grew from the *P. protegens*-treated fruits and the control fruits on the first day. In the subsequent days, the number of the mycelial cluster increased over time. On the fifth day, there were 46.67 mycelial clusters on the control fruits, while only 11.67 on the *P. protegens*-treated fruits, which was decreased by 72.00% compared to the control (Figure 4A). On the seventh day, the disease spot emerged from both the treated and the control fruits, but the size of the disease spots on the *P. protegens*-treated fruits (Figure 4B) was smaller than the control (Figure 4C). The number of disease spots on the control fruits averaged 5.00, whereas 12.33 on the *P. protegens*-treated fruits, 56.46% smaller than the control (Figure 4D). On the ninth day, the disease spots on the control fruits expanded and joined together, forming a large area of rotten tissues. However, there were only 8.70 separated disease spots on the *P. protegens*-treated fruits, denoting that the *P. protegens* substantially inhibited the apple ring disease.

### *Pseudomonas protegens* improved the fruit quality of the postharvest apple fruit to some extent

**Figure 5:**
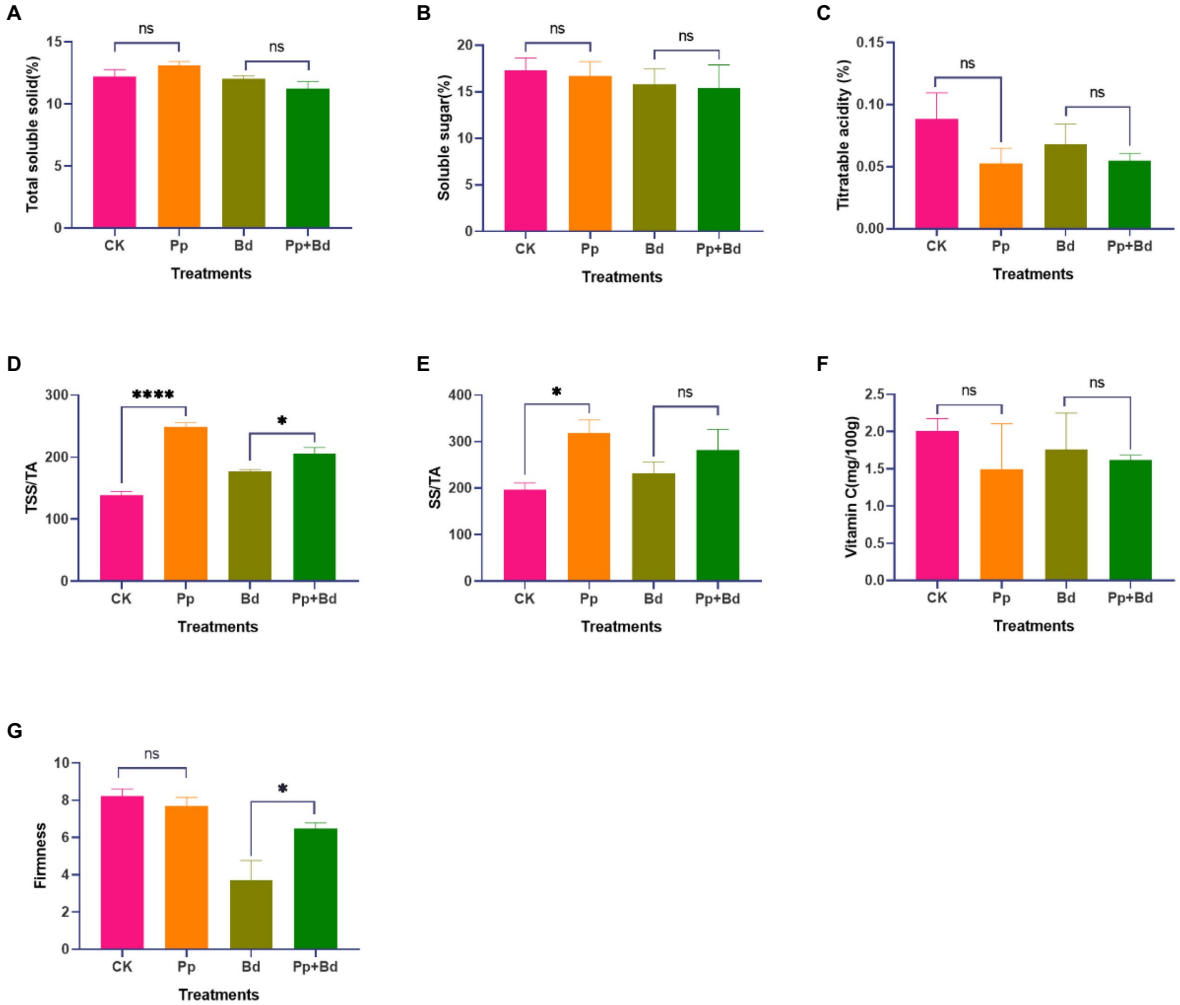
The fruit quality indexes of the apple fruits of the four treatments. Nine days later, the fruit quality indexes, including total soluble solid (TSS) **(A)**, titratable acidity (TA) **(B)**, soluble sugar (SS) **(C)**, total soluble solid/titrable acidity (TSS/TA) **(D)**, and soluble sugar/titrable acidity (SS/TA) **(E)**, vitamin C (VC) **(F)**, and firmness **(G)**, were different among the four treatment. ^*^p < 0.05, ^****^p < 0.0001, ns *p* > 0.05.

On the ninth day, we compared the fruit quality indexes, including the TSS, SS, TA, VC, TSS/TA, SS/TA, and the firmness of apple fruits from the four treatments, to evaluate the effect of the *P. protegens* on fruit quality (Figure 5). *P. protegens* significantly improved TSS/TA ratio and SS/TA ratio in the apple fruits without inoculating with *B. dothidea*. Compared to the control (CK), the TSS/TA ratio and SS/TA ratio in the *P. protegens*-treated fruits (Pp) were enhanced by 80.67 and 61.90%, respectively. In addition, the *P. protegens* decreased the TA and VC content by 40.51 and 25.24%, but statistics showed the difference was non-significant. However, *P. protegens* significantly increased the TSS/TA ratio and fruit firmness of the apple fruit inoculated with *B. dothidea*. The TSS/TA ratio and the firmness of the *P. protegens*-treated fruits (Pp + Bd) were 16.11 and 74.03% higher than the control (Bd). Meanwhile, *P. protegens* caused an apparent but non-significant alternation in the SS/TA ratio and TA content, increasing by 21.40% and decreasing by 19.68%, respectively, compared to the control.

### *Pseudomonas protegens* significantly induced the expressions of defense-related genes in apple fruits

**Figure 6:**
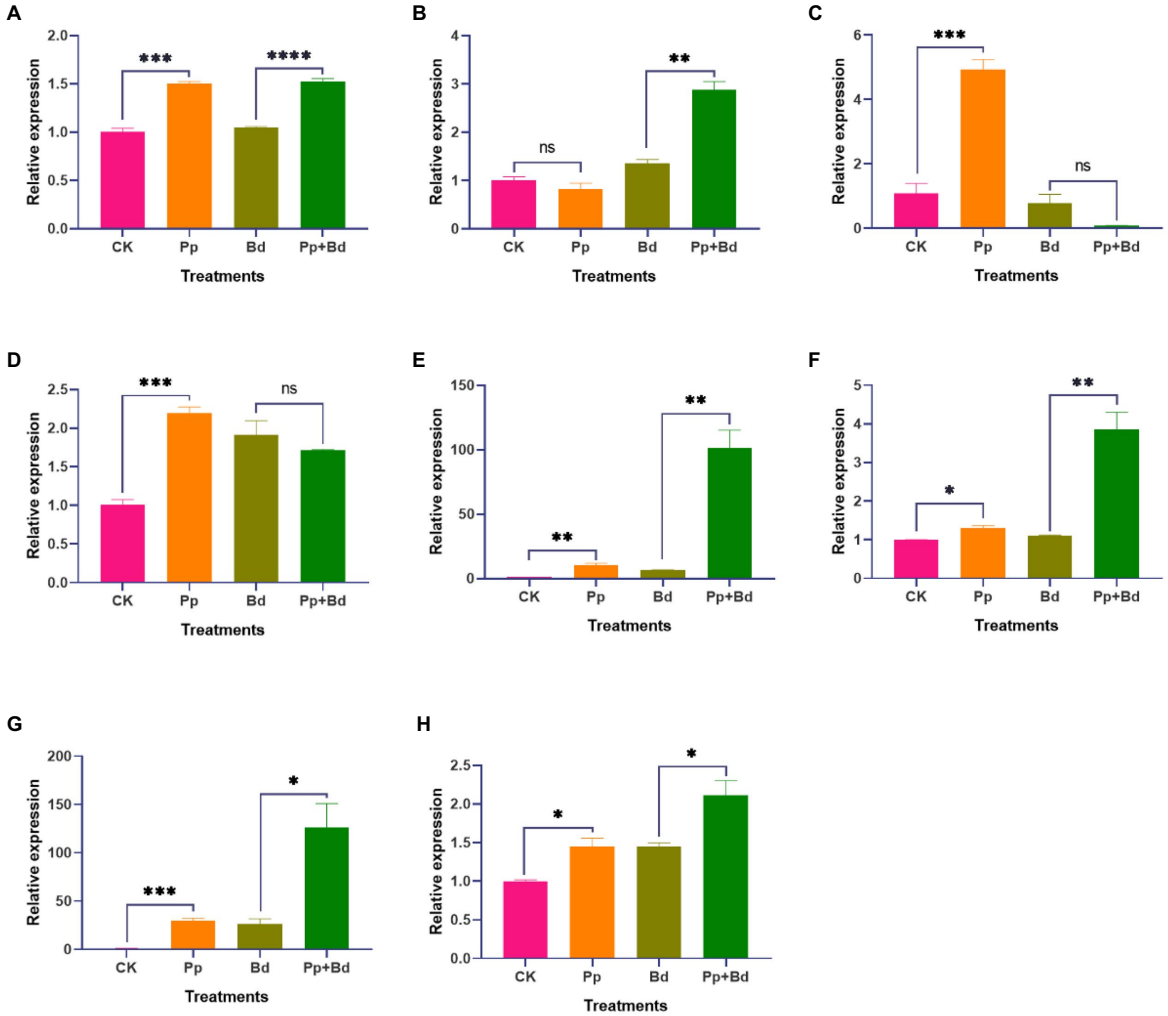
The relative expressions of various defense-related genes and the transcription factors, including MdGLU **(A)**, MdCATe **(B)**, MdPOD **(C)**, MdPAL **(D)**, MdPUB29 **(E)**, MdWRKY15 **(F)**, MdERF11 **(G)** and MdMYB73 **(H)** in various treated apple fruits. ^*^p < 0.05, ^**^< 0.01, ^***^p < 0.001, ^****^p < 0.0001, ns *p* > 0.05.

Whether the apple fruits were infected with the pathogen fungus *B. dothidea*, *P. protegens* considerably induced the expressions of defense-related genes such as MdGLU, MdPOD, MdCAT, and MdPAL in apple fruits (Figures 6A–D). In the case of *B. dothidea* infection, the MdGLU, MdPOD, and MdPAL in apple fruits treated with *P. protegens* (Pp + Bd) were 50.00, 357.07, 118.64% higher than the control (Bd). However, in the absence of *B. dothidea*, the MdGLU and MdCAT in *P. protegens*-treated apple fruits (Pp) were increased by 45.44 and 111.53% compared with control (CK).

In addition, *P. protegens* drastically induced the expressions of the transcription factors related to the apple fruit resistance against *B. dothidea*, including the MdPBU29, MdWRKY15, MdERF11, and MdMYB73, regardless of whether the apple fruit was infected with *B. dothidea* or not (Figures 6E–H). Compared to the control (CK), the expressions of these genes in *P. protegens*-treated apple fruits (Pp) were increased by 920.89, 2778.54, 44.81, and 30.14% in the absence of *B. dothidea* infection. While in the presence of *B. dothidea* infection, the expressions of these genes in *P. protegens*-treated apple fruits (Pp + Bd) were 1480.74, 374.78, 45.40, and 249.57% higher than the control (Bd).

### *Pseudomonas protegens* significantly decreased the expressions of pathogenicity-related genes in *Botryosphaeria dothidea*

**Figure 7:**
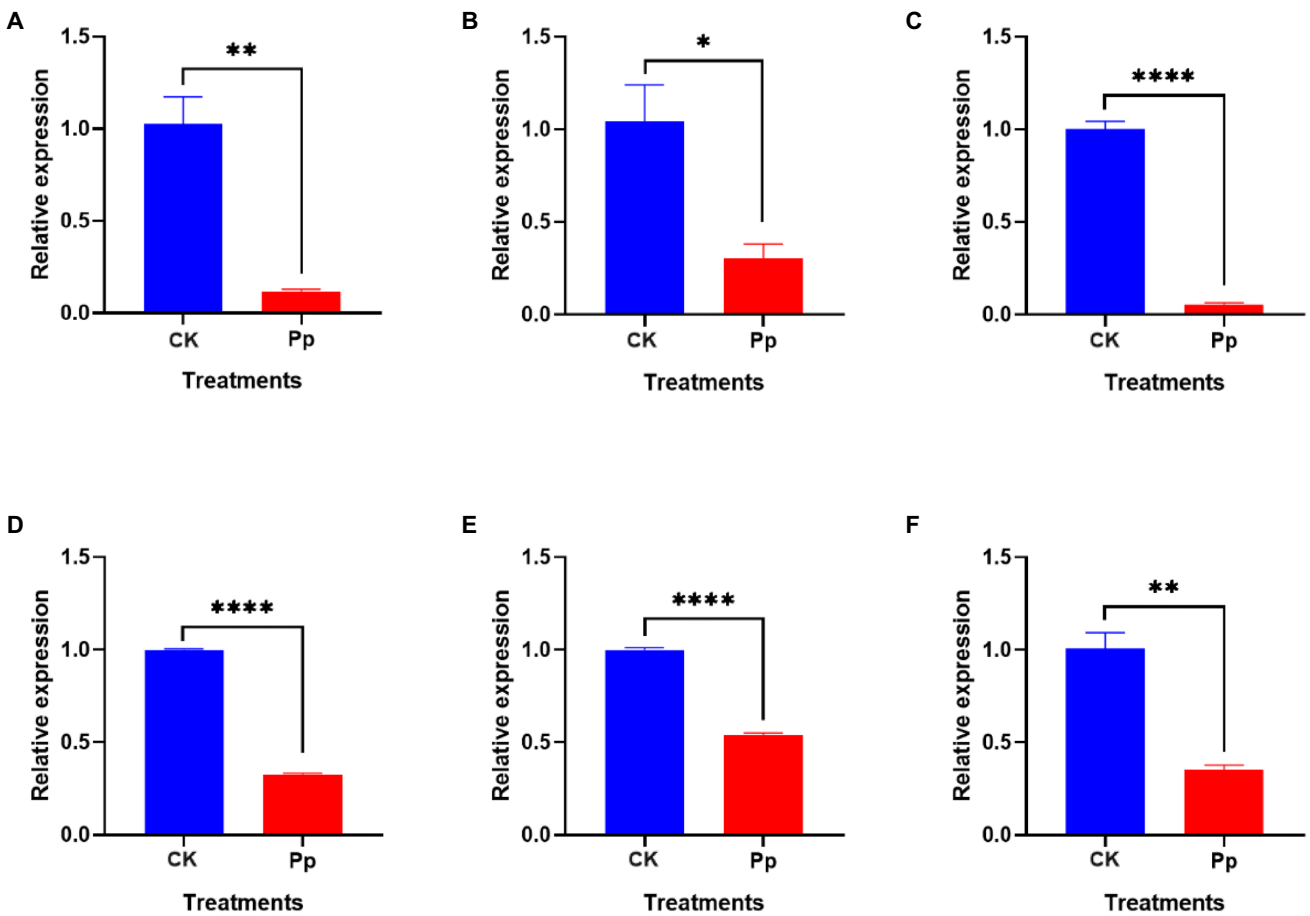
The relative expressions of various pathogenicity-related genes, including BdCYP450 **(A)**, BdADH **(B)**, BdGHY **(C)**, BdATS **(D)**, Bdα/β-HY **(E)**, and BdSTR **(F)** in *P. protegens*-treated and the control *B. dothidea*. ^*^p < 0.05, ^**^p < 0.01, ^****^p < 0.0001.

To determine the inhibitory effect of *P. protegens* on the *B. dothidea*, we tested the expressions of genes related to fungal pathogenicity, including BdCYP450, BdADH, BdGHY, BdATS, Bdα/β-HY, and BdSTR (Figure 7). The analysis revealed that the relative expressions of these genes in *B. dothidea* mycelia treated with the *P. protegens* (Pp) were reduced by 88.47, 70.90, 94.34, 67.38, 46.03, and 64.88% compared to the control (CK) 6 h later, which indicated that the *P. protegens* significantly repressed the pathogenicity-related genes in *B. dothidea*.

## Discussion

Our preliminary studies showed that intercropping or rotating with Chinese leek (*Allium tuberosum*) dramatically decreased the incidence and severity of banana *Fusarium* wilt caused by *Fusarium oxysporum* f. sp. *cubense* race 4 (Foc4) (Huang et al., 2012). Furthermore, tomato or cucumber intercropped or rotated with Chinese leek significantly reduced root-knot nematode disease (Huang et al., 2016). In addition, we also found that Chinese leek extract (Zhao et al., 2017) and volatiles (Fu et al., 2022) markedly suppressed the apple ring rot on postharvest fruits. Given that fact, we inferred that there were components or endophytic bacterium with antifungal activity in Chinese leek plants. Fortunately, we corroborated that the dimethyl trisulfide, one of the main components of Chinese leek volatiles (Sun et al., 2022a), and the endophytic bacterium *Serratia plymuthica* isolated from Chinese leek (Sun et al., 2022b), showed strong inhibitory effect against *B. dothidea*. In the present study, we validated that another endophytic bacterium *P. protegens*, isolated from Chinese leek, had a significant inhibitory effect on *B. dothidea* and strikingly reduced the apple ring rot on postharvest fruits. These antifungal components and endophytic bacterium were the major factors contributing to the efficient control of Chinese leek on apple ring disease.

Our present study manifested that *P. protegens* was a promising biocontrol agent to inhibit *B. dothidea*, concordant with numerous earlier studies (Jing et al., 2020; Zhang et al., 2020; Pellicciaro et al., 2021; Prigigallo et al., 2021). However, the suppression of *P. protegens* against *B. dothidea* on the PDA medium was substantially lower than that in the PDB medium. The main possible reasons were that there was a distance between *P. protegens* and *B. dothidea* when they were just inoculated on the PDA medium. They contacted and interacted with each other at a later time. At this time, *B. dothidea* had already exhibited vigorous growth, and only the mycelial apex reached the *P. protegens*, limiting the antifungal activity of *P. protegens*. However, *P. protegens* and *B. dothidea* were fully contacted and interacted with each other at the first moment when they were inoculated in the PDB medium. What is more, *B. dothidea* did not thrive at that moment. Consequently, *P. protegens* quickly suppressed the growth of *B. dothidea*. As a result, all these factors brought about the higher repression of *P. protegens* on *B. dothidea* in the PDB medium than in the PDA medium.

Due to the suppression of *P. protegens* on the growth of *B. dothidea*, *P. protegens* showed significant inhibition against apple ring rot on postharvest fruits. We artificially wounded and inoculated the apple fruits with the pathogen fungal disc in the experiment. Generally, in practical production, almost all the apple fruit were intact and not necessarily infected with the fungus. Moreover, the fungal amount contaminating fruits in the field was less than that we inoculated in the laboratory. Accordingly, the *P. protegens* would have a higher inhibitory effect on the disease in actual production. More importantly, *P. protegens* also improved the fruit quality to different extents, killing two birds with one stone.

Our study demonstrated that the *P. protegens* significantly induced the MdGLU, MdPAL, MdPOD, and MdCAT expressions in apple fruit. Previous studies showed that these genes participated in plant resistance to various pathogens. For example, GLU genes in *Panax notoginseng* (Taif et al., 2020) and soybean (Glycine max) (Shi et al., 2020) were vital defense genes against *Fusarium solani* and soybean mosaic virus infection. PAL gene in *Lotus japonicus* (Chen et al., 2017) and soybean (Zhang et al., 2017) affected rhizobial infection progress and resistance to *Phytophthora sojae*. POD gene in potatoes (Yang Y. et al., 2020) and sweet oranges (Li et al., 2020) enhanced the resistance against *Phytophthora infestans*, and *Xanthomonas citri* subsp. *citri* (Li et al., 2020). CAT genes in maize (Jiao et al., 2021) and *Nicotiana tabacum* contributed to resistance against maize chlorotic mottle virus and chili veinal mottle virus infection (Yang T. et al., 2020). In addition, *P. protegens* also significantly induced several transcription factor expressions in apple fruits, including MdERF11, MdMYB73, MdPUB29, and MdWRKY15. Previous studies showed that overexpression of the MdERF11 (Wang et al., 2020), MdMYB73 (Gu et al., 2021), MdPUB29 (Han et al., 2019), and MdWRKY15 (Zhao et al., 2020) in apple fruits significantly increased the fruit resistance to *B. dothidea* infection, whereas silencing these genes in apple fruits resulted in decreased resistance. Therefore, the increased expressions of these genes in apple fruits may be one of the important factors contributing to the disease reduction.

We also found that *P. protegens* significantly repressed the BdCYP450, BdADH, BdGHY, BdATS, Bdα/β-HY, and BdSTR expressions in *B. dothidea*. Previous studies revealed that these genes were involved in fungal development and pathogenicity. For instance, Cytochrome P450s (CYP450) participated in the virulence of *Fusarium graminearum* (Shin et al., 2017) and *Verticillium dahlia* (Zhang et al., 2016). Alcohol dehydrogenase (ADH) was required for fungal development, environmental adaptation, and its ability for full pathogenicity in *Botrytis cinerea* (DafaAlla et al., 2021). Glycoside hydrolase (GHY) functioned as an important determinant of virulence in *Coniella vitis* (Qin et al., 2020), *Phytophthora sojae* (Ma et al., 2015), and *Pyricularia oryzae* (Pan et al., 2021). Aminoacyl-tRNA synthetase (ATS) was a vital factor in the cellular viability of *Ustilago maydis* (Ostrowski and Saville, 2017) and germination and blastospore yield in *Beauveria bassiana* (Zhu et al., 2017). Alpha/beta hydrolase (α/β-HY) was involved in the *Fusarium graminearum* pathogenicity (Jiao and Peng, 2018). Sugar Transporter (STR) played an essential role in the germination and mycelial growth of *Metarhizium robertsii* (Dai et al., 2021). From this, the significantly reduced expression of these genes in *B. dothidea* was another important factor contributing to the disease reduction.

**Figure 8:**
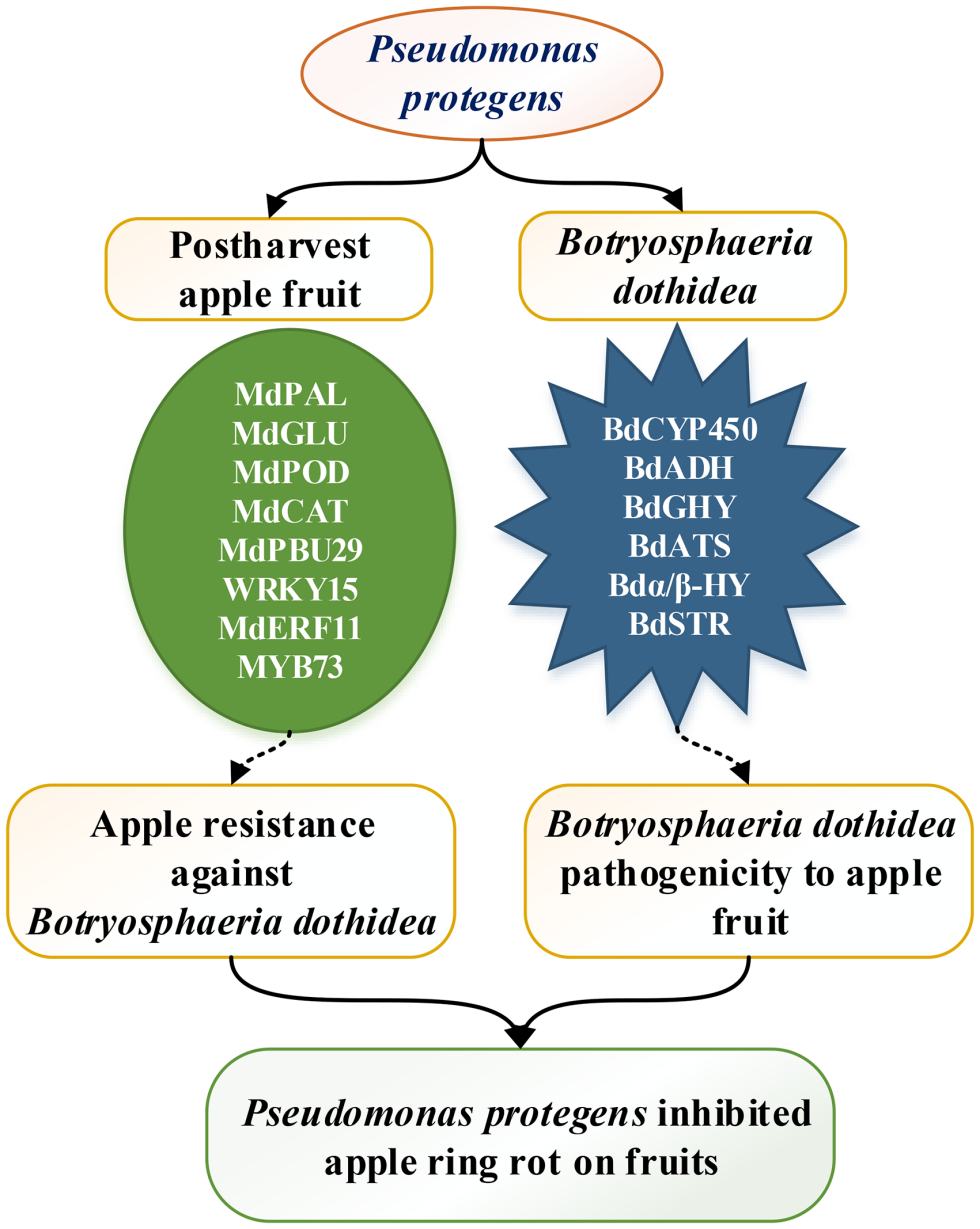
The model of *P. protegens* inhibiting the apple ring rot on fruit caused by *B. dothidea*.

Taken together, the molecular mechanism by which *P. protegens* significantly inhibited the apple ring rot on postharvest apple fruit can be explained from two aspects (Figure 8). On the one hand, *P. protegens* induced the defense-related genes, including MdPAL, MdGLU, MdPOD, and MdCAT in apple fruits, and the transcription factors such as MdPUB29, MdWRKY15, MdEFR11, and MdMYB73, which enhanced the apple fruit resistance against pathogen *B. dothidea*. On the other hand, *P. protegens* strongly repressed the pathogenicity-related genes in *B. dothidea*, such as BdCYP450, BdADH, BdGHY, BdATS, Bdα/β-HY, and BdSTR, which reduced the pathogenicity of *B. dothidea* to apple fruits. The two factors, together, contributed to the considerable reduction of the ring rot disease on *P. protegens*-treated apple fruits.

## Conclusion

*P. protegens* isolated from Chinese leek strongly suppressed the mycelial growth of the pathogen *B. dothidea*, thus further significantly inhibiting the apple ring rot on postharvest apple fruits. Simultaneously, *P. protegens* also improved the fruit quality to some extent. Furthermore, *P. protegens* significantly induced the defense-related genes in apple fruits and markedly repressed the pathogenicity-related genes in *B. dothidea*. Together, the two-fold factors led to the vast reduction of apple ring rot disease on postharvest apple fruits.

## Data availability statement

The original contributions presented in the study are included in the article/Supplementary material, further inquiries can be directed to the corresponding authors.

## Author contributions

YH and YD contributed to the conception of the study and wrote and reviewed the manuscript. JpL, JhL, and XS experimented and collected the data. All authors contributed to the article and approved the submitted version.

## Funding

This work was supported by the National Natural Science Foundation of China (31471864), the Natural Science Foundation of Shandong Province (ZR2020MC143 and ZR2020MC136), the Agricultural Variety Improvement Project of Shandong Province 2020LZGC007, and the Qingdao Agricultural University High-level Personnel Startup Fund China (6631115024).

## Conflict of interest

The authors declare that the research was conducted in the absence of any commercial or financial relationships that could be construed as a potential conflict of interest.

## Publisher’s note

All claims expressed in this article are solely those of the authors and do not necessarily represent those of their affiliated organizations, or those of the publisher, the editors and the reviewers. Any product that may be evaluated in this article, or claim that may be made by its manufacturer, is not guaranteed or endorsed by the publisher.

## Footnotes

^1^ https://www.fao.org/faostat/zh/#data/QCL

^2^ https://www.ncbi.nlm.nih.gov/
